# CD8 + T cell infiltration is associated with improved survival and negatively correlates with hypoxia in clear cell ovarian cancer

**DOI:** 10.1038/s41598-023-30655-3

**Published:** 2023-04-21

**Authors:** Nancy Guo, Aijun Yang, Fabiha Binte Farooq, Shreena Kalaria, Elena Moss, Lindsay DeVorkin, Mary Lesperance, François Bénard, Don Wilson, Anna V. Tinker, Farouk S. Nathoo, Phineas T. Hamilton, Julian J. Lum

**Affiliations:** 1 Department of Mathematics and Statistics, University of Victoria, STN CSC, PO BOX 1700, Victoria, BC V8W 2Y2 Canada; 2 Department of Economics, University of Victoria, Victoria, Canada; 3 Trev and Joyce Deeley Research Centre, BC Cancer, Victoria, 2410 Lee Avenue, 3rd Floor, Victoria, BC V8R 6V5 Canada; 4 Department of Radiology, University of British Columbia, Vancouver, Canada; 5 Functional Imaging, BC Cancer Vancouver, Vancouver, Canada; 6 Medical Oncology, BC Cancer Vancouver, Vancouver, Canada; 7 Department of Biochemistry and Microbiology, University of Victoria, Victoria, Canada

**Keywords:** Tumour biomarkers, Tumour immunology, Immunotherapy, Statistics, Biomarkers

## Abstract

Unlike other histological types of epithelial ovarian carcinoma, clear cell ovarian carcinoma (CCOC) has poor response to therapy. In many other carcinomas, expression of the hypoxia-related enzyme Carbonic anhydrase IX (CAIX) by cancer cells is associated with poor prognosis, while the presence of CD8 + tumor-infiltrating lymphocytes (TIL) is positively prognostic. We employed [^18^F]EF5-PET/CT imaging, transcriptome profiling, and spatially-resolved histological analysis to evaluate relationships between CAIX, CD8, and survival in CCOC. Tissue microarrays (TMAs) were evaluated for 218 cases in the Canadian COEUR study. Non-spatial relationships between CAIX and CD8 were investigated using Spearman rank correlation, negative binomial regression and gene set enrichment analysis. Spatial relationships at the cell level were investigated using the cross K-function. Survival analysis was used to assess the relationship of CAIX and CD8 with patient survival for 154 cases. CD8 + T cell infiltration positively predicted survival with estimated hazard ratio 0.974 (95% CI 0.950, 1000). The negative binomial regression analysis found a strong TMA effect (p-value < 0.0001). It also indicated a negative association between CD8 and CAIX overall (p-value = 0.0171) and in stroma (p-value = 0.0050) but not in tumor (p-value = 0.173). Examination of the spatial association between the locations of CD8 + T cells and CAIX cells found a significant amount of heterogeneity in the first TMA, while in the second TMA there was a clear signal indicating negative spatial association in stromal regions. These results suggest that hypoxia may contribute to immune exclusion, primarily mediated by effects in stroma.

## Introduction

Dysregulation of metabolism in the tumor microenvironment (TME) is a hallmark of cancer. As a metabolic process, hypoxia can contribute to malignant phenotypes including aggressiveness, metastases, and immune suppression^1–3^. At present, there are three main modalities to assess hypoxia in human cancer patients; nuclear imaging using PET/CT with nitroimidazole compounds such as EF5 or pimonidazole, immunostaining with markers such as hypoxia inducible factor 1 alpha (HIF-1α), downstream HIF-1α targets (e.g. CAIX, VEGF), and transcriptomic analyses based on gene expression signatures of hypoxia^4^. Indeed, several pre-clinical and clinical reports have found that hypoxia markers correlate with fewer tumor-infiltrating lymphocytes and poor patient outcomes^5–10^. Evidence from in vitro and preclinical mouse models suggests that hypoxic tumors have an extrinsic role in repressing antitumor immunity by suppressing cytotoxic T lymphocyte (CTL)-mediated tumor killing, either by excluding CTLs, affecting their cytolytic capacities, or supporting T regulatory cell function^5,11^. Reduced oxygen levels could therefore serve as a barrier to both endogenous immune responses as well as those produced by immunotherapy treatments such as adoptively transferred T cells or chimeric antigen receptor T cells (CAR-T)^12^. However, studies in genetically engineered mouse models found that HIF-1α or deletion of the Von Hippel-Lindau factor could promote enhanced differentiation of tissue-resident memory-like T cells with higher poly-functionality^7,13,14^. These results suggest that there may exist intrinsic differences between the HIF response pathway’s influence and that of oxygen levels on T cell behavior in the TME.

Clear cell ovarian carcinoma (CCOC) is one of five major ovarian cancer histological subtypes and represents 10–15% of ovarian cancer cases. While often diagnosed at early stages, more widespread or recurrent CCOC is associated with poor outcomes and represents a therapeutic challenge. Chemotherapy response rates tend to be low and treatment benefits are brief^15^. Drug resistance develops early and patients with advanced CCOC have worse outcomes than the common high grade serous tubo-ovarian carcinoma^16^. Unlike a majority of carcinomas, studies in CCOC have not found infiltrating T cells to associate with improved overall survival^17^. Whether the resistance or sensitivity to treatment is associated with hypoxia or TIL is unclear. At present, most human studies have employed single-marker analysis of hypoxia and TIL classifications. As hypoxia has been associated with impaired antitumor immunity in diverse cancers, we reasoned that low oxygen may reduce or impair T cell infiltration to compromise antitumor immunity in CCOC.

Here, a multiple platform approach using [^18^F]EF5-PET/CT, transcriptomic analysis, and immunostaining was employed to assess whether tumors in patients with CCOC demonstrate evidence of hypoxia and how this might associate with CD8 + TIL infiltration. This allowed us to test (i) the utility of these strategies to detect tumor hypoxia, (ii) whether markers of hypoxia, in patients predicted reduced CD8 + T cell infiltration, and (iii) whether micro-regional variation in CAIX expression predicted T cell exclusion from subregions of the TME. Specifically, associations between CAIX and CD8 + T cells were analyzed by adopting statistics of co-occurrence of point patterns from landscape ecology and spatial statistics. Clear associations with CD8 + and CAIX were observed as well as spatial interactions depending on tumor or stroma expression. In contrast to the presence of CD8 + T cells, CAIX alone was not found to be associated with survival. Overall, hypoxia is a feature of CCOC and this was to have a negative association with CD8 + in the stroma regions of the tumor.

## Material and methods

### [^18^F]EF5-PET/CT imaging study in CCOC patients

This study was conducted at BC Cancer Vancouver following approval by the BC Cancer Research Ethics Board (H13-00921). All patients provided written informed consent (NCT01881451). All methods were performed in accordance with relevant guidelines and regulations. Inclusion criteria: histologically confirmed, advanced metastatic or recurrent clear cell cancer of the ovary, at least one index lesion measuring 2 cm in diameter, and not on active therapy for ≥ 4 weeks. Participants underwent one EF5-PET/CT scan. A bolus intravenous dose of 185 MBq (5 mCi) to 370 MBq (10 mCi) of [^18^F]EF5 was delivered, followed by a 10–20 mL normal saline (NS) flush.

### Uptake of 18F-EF5

18F-EF5 uptake was evaluated semi-quantitatively by determining the tumor-to-muscle activity ratio (T/M). Standardized uptake values (SUV) were calculated for suspicious areas using a region of interest drawn around the target area on the PET images where SUV = (peak activity/mL in region of interest) / (injected activity/g of body weight). A Tumor-to-muscle ratio of > 1.5 was considered positive.

### Tissue cohort, staining and data acquisition

Diagnostic tissues from patients participating in the [^18^F]EF5-PET/CT imaging study were obtained from the Cheryl Brown Outcomes Unit. Tumor tissue microarrays (TMAs) were obtained from the Canadian Ovarian Experimental Unified Outcomes Resource (COEUR), a Canadian-compiled resource for interrogation of ovarian cancer subtypes^18^ with approval from the UBC-BC Cancer Research Ethics Board (Certificate: H20-02211). Provided TMAs had been constructed from small core biopsies (0.7 mm) of representative areas of tumor tissues. For staining, slides were deparaffinized with xylene (Fisher Scientific) and rinsed with dH2O. Antigen retrieval was performed in Diva Decloaking Solution (Biocare Medical, USA). Staining was performed on an intelliPATH platform (Biocare Medical, USA) with the following antibodies: CAIX (clone M75, catalogue number AB1001, dilution: 1/50, Bioscience Slovakia; kind gift from Dr. Peter Watson) and CD8 (clone 4SM15, catalogue number 14-0808-82, dilution 1/250, eBioscience™). Slides were air-dried and cover-slipped with ecomount (Biocare Medical, USA). The majority of the participants had two TMA cores, with the cores from each subject being on one of two TMAs (hereafter TMA A and TMA B). Whole slide images were scanned (Panoramic Midi) and CAIX and CD8 expressing cells scored using the software QuPath^19^. (Multiple digital pathology methods were applied to quantify CAIX and CD8 staining, including QuPath and Akoya inForm; QuPath was selected as the best performing method after qualitative review of classifications by multiple parties.) Cells were also classified as stromal or epithelial cells. After manual inspection to remove cores with inadequate tumor tissue or staining artifacts, TMA A provided 183 cores and TMA B 218 cores. While no blinded evaluation of classifier performance was performed, positive classifications were highly concordant with positivity as evaluated by an experienced image analyst. Overall CD8 and CAIX cell densities for each participant were calculated using the number of CD8 or CAIX cells divided by the area of the core. In addition, tumor and stromal cell densities were calculated by using the number of CD8 and CAIX cells from the respective tissue types divided by the corresponding tumor or stromal area. Cells were not phenotyped beyond CD8 positivity and tumor/stroma classifications so any detected cell classified as CAIX + was counted as CAIX + . In practice, CAIX expression appeared to be primarily on tumor cells in tumor epithelial regions and fibroblasts in stromal regions. However, we cannot rule out other CAIX-expressing cell types (e.g. macrophages) due to a lack of additional markers in the panel to unambiguously resolve additional cell types.

### Clinical characteristics of the COEUR cohort

Age at diagnosis, family history of cancer, tumor grade (1 = well differentiated, 2 = moderately differentiated, 3 = poorly differentiated, and NA), FIGO tumor stage (I, II, III and IV), and debulking were evaluable. Debulking (1 = Yes or 0 = No) refers to residual disease present after first debulking/staging surgery, where level 0 includes macroscopic disease none, <  = 1 cm, and optimal; and level 1 includes macroscopic disease 1–2 cm, > 2 cm, miliary and suboptimal. The follow-up time in months along with vital status was used as a censored overall survival response. For the survival and clinical information there were a total of 218 subjects. After removing subjects with data inconsistencies or missing covariates (primarily in age and FIGO tumor stage), 179 had evaluable TMA tissue cores. We further required that > 100 cells were detected and scored to include each image, resulting in 154 participants with linked clinical and TMA tissue cores for survival analysis. For the core level analysis of the spatial distribution and relationship between CD8 T cells and CAIX we had 183 (TMA A) and 218 (TMA B) cores.

### Transcriptomics analysis

Four publicly available cohorts were used for transcriptomics studies: GSE29450^20^ consisting of gene expression profiling from 10 CCOC specimens and 10 samples of normal ovarian surface epithelium, GSE6008^21^ consisting of 8 CCOC specimens and 4 individual normal ovary samples, GSE73614^22^ consisting of 36 CCOC specimens, and GSE129617^23^ consisting of 23 CCOC specimens. Single-sample Gene Set Enrichment Analysis (ssGSEA) was used to calculate gene set or pathway scores on a per-sample basis^24^ and a predetermined gene sets (from MsigDB^25^) to obtain a hypoxia score. A Spearman Rank correlation test between log CD8A expression and the hypoxia score and CAIX expression alone was used for each study.

### Spatial analysis of CAIX and CD8 + T cells

For each patient, we compared the counts of CAIX-expressing cells and CD8 + T cells (normalized as cells/ μm^2^) using Spearman correlation and the nonparametric bootstrap to obtain confidence intervals. Spatial analyses were performed using the locations of individual cells as well as the cell type on a given core yielding a multivariate (marked) spatial point pattern, and examined relationships between different types of points, namely, CAIX and CD8 expressing cells. The cross-K function^26^ represents this relationship and is defined as

$$ K_{ij} (d) = \frac{{E({\text{number}}\,\,{\text{of}}\,\,{\text{type}}\,\,j\,\,{\text{point}}\,\,{\text{within}}\,\,{\text{a}}\,{\text{radius}}\,\,d\,\,{\text{of}}\,\,{\text{a}}\,\,{\text{type}}\,\,i\,\,{\text{point}})}}{{\lambda_{j} }} $$

where $E()$ denotes the expected value under the processes generating the locations of CAIX and CD8 cells and ${\lambda }_{j}$ represents the intensity of points of type j. The estimated cross-K function was compared to its theoretical form (${K}_{ij}(d) = \pi {d}^{2}$) under the null to determine deviations from independence at different distances within tissue cores. For each core we also obtained a 99% Monte Carlo envelope corresponding to independence for each core, using the R package *spatstat*^27^. In some cases, the data were too sparse such that there was not enough spatial information to achieve stability of the envelope. We thus computed the cross-K function for cores with cell density greater than $4\times {10}^{-5}$ cells/µm^2^. A family-wise Monte Carlo envelope was then obtained by aggregating the 99% simulation envelopes for all evaluable cores by taking the minimum and maximum over all envelopes for each distance.

### Tumor and stroma core level analysis of CAIX and CD8 + T cells

To examine the association between CAIX and CD8 at the core level we computed the density of CAIX and CD8 cells in each core, both overall and restricted to either the tumor or stromal tissues. We quantified this association using the Spearman correlation along with 95% bootstrap confidence intervals. To better account for the discrete nature of the data as well as to allow for a more general form of association than the Spearman correlation, we used regression models for count data that allow for potential slide-specific (e.g. batch) effects through an interaction term between TMA slide and CAIX. A negative binomial regression and zero-inflated Poisson regression was used for data analysis^28^.

### Survival analysis

Kaplan–Meier survival curves^29^ stratified by various categorical variables were generated and the corresponding groups compared using the log-rank test. Univariable and multivariable Cox proportional hazard regression models were fit to determine associations between survival and demographic and clinical characteristics, and CAIX and CD8 density. The analysis was carried using the average of CD8 and CAIX density across multiple cores (2–3 cores) for each participant. We assessed model adequacy by checking martingale and deviance residuals plots, examined linear functional forms of continuous covariates, and checked the proportional hazards assumption^30^. All p-values were based on 2-sided hypothesis tests and the statistical analyses were performed using SAS 9.4 (SAS Institute Inc.)^31^.

### Ethics approval

The [^18^F]EF5-PET/CT imaging study in CCOC patients was conducted at BC Cancer Vancouver and approved by the University of British Columbia and BC Cancer Research Ethics Board (H13-00921). All patients provided written informed consent (NCT01881451). Tissues were obtained from the Cheryl Brown Outcomes Unit. Tumor tissue microarrays (TMAs) were obtained from the Canadian Ovarian Experimental Unified Outcomes Resource (COEUR), a Canadian-compiled resource for interrogation of ovarian cancer subtypes^18^ with approval from the UBC-BC Cancer Research Ethics Board (Certificate: H20-02211).

## Results

### Detection of [^18^F]EF5-PET positive tumor lesions

**Figure 1:**
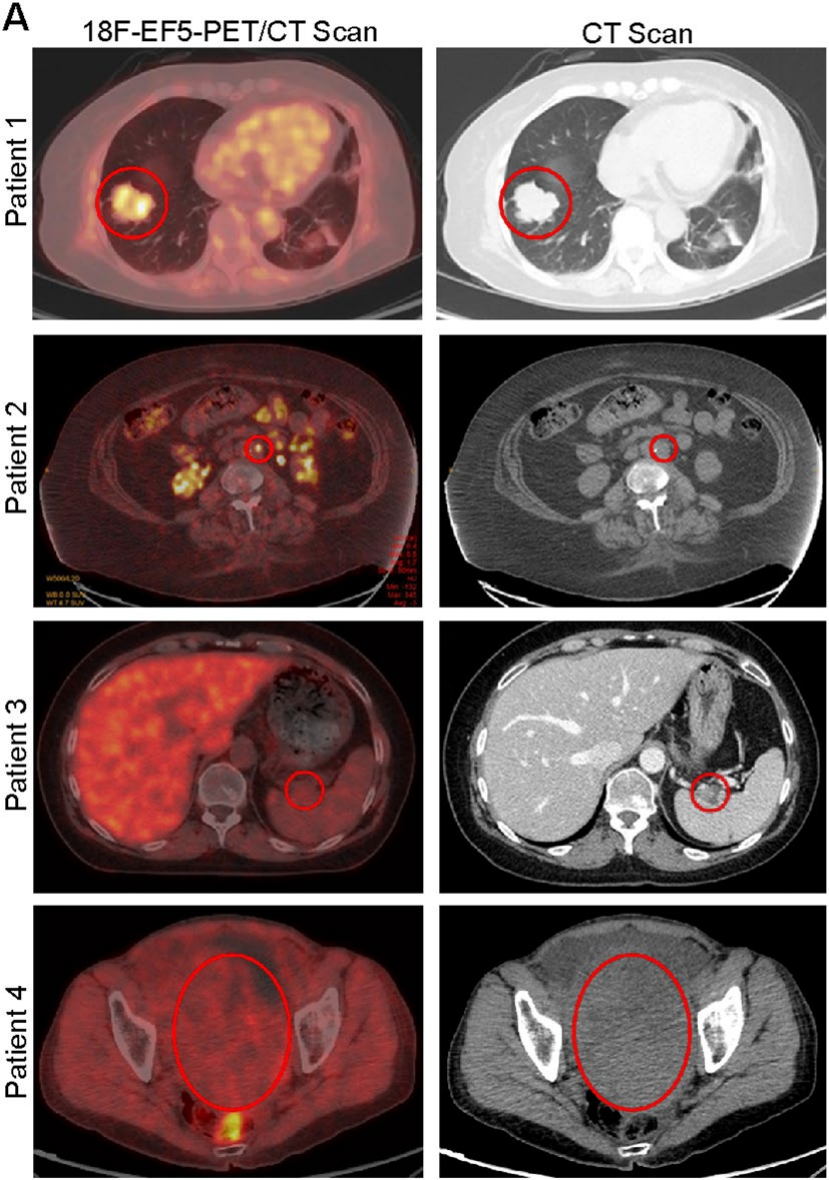
[^18^F]EF5-PET/CT positivity in tumors from CCOC patients. Representative images from each of the four CCOC patients. In two cases, patient 1 and patient 2, detectable EF5 positive was observed.

At present, tumor hypoxia in CCOC has not been formally demonstrated in situ, although some studies suggest that tissue markers of hypoxia from surgically resected CCOC tumors show evidence of hypoxia^32–36^. A small pilot [^18^F]EF5-PET/CT imaging study was conducted in recurrent platinum sensitive CCOC patients to determine whether tumor hypoxia could be detected. In total, four patients were enrolled on the study. Of these, two patients had tumors that had measurable levels of EF5 uptake (Fig. 1). Many small tumor lesions had evidence of EF5 uptake. Taken together, this suggests that tumor hypoxia is a feature attributed to some CCOC patients.

### Hypoxia transcriptional signatures and association with TIL

Hypoxia gene signatures have been widely used to assess the state of hypoxia in tumor tissue^37,38^. To examine the relationship between hypoxia and immune cell associations, we analyzed four publicly available ovarian cancer cohorts for expression of Molecular Signature Database (MSigDB)^39^ genes, as a proxy for a hypoxia score^40,41^ and *CD8A* as a marker for infiltration of T cells. The genes used to construct the hypoxia score are listed in Supplemental Table S8. In each cohort, we selected only the CCOC histotype with a total of 77 cases. A Spearman correlation analysis was conducted on the relationship between Log-*CD8A* and the hypoxia score (Table 1). Based on this test and the p-values indicated in the first row of Table 1, there was no evidence for the association between CD8 expression and hypoxia across the four different cohorts (Supplemental Fig. S1). We also analyzed whether carbonic anhydrase IX (CAIX), a transcriptional target of the hypoxia-inducible factor-1 (HIF-1) could be used to stratify the presence or absence of CD8. From the value of the correlation coefficients as well as the scatterplots there was strong detectable relationship between CAIX and CD8 in two cohorts (Supplemental Fig. S2 and the second row of Table 1; GSE73614, $\rho$ = 0.48, p = 0.003; GSE129617, $\rho$ = 0.70, p < 0.001). In contrast, the coefficients for this analysis were estimated as negative for the GSE29450 and GSE6008 CCOC cohorts.

**Table 1:** Spearman rank correlation test for the relationship between Log-CD8A expression and hypoxia score (first row) and CAIX (second row) obtained from four NCBI studies.

	GSE29450 (n = 10)		GSE6008 (n = 8)		GSE73614 (n = 36)		GSE129617 (n = 25)	
	$\rho$	P-value	$\rho$	P-value	$\rho$	P-value	$\rho$	P-value
Hypoxia	0.20	0.58	0.024	0.98	0.022	0.90	− 0.25	0.23
CAIX	− 0.15	0.68	− 0.14	0.75	0.48	0.003	0.70	< 0.001

### Tumor and stroma differences in hypoxia and association with CD8 + T cell infiltration

**Figure 2:**
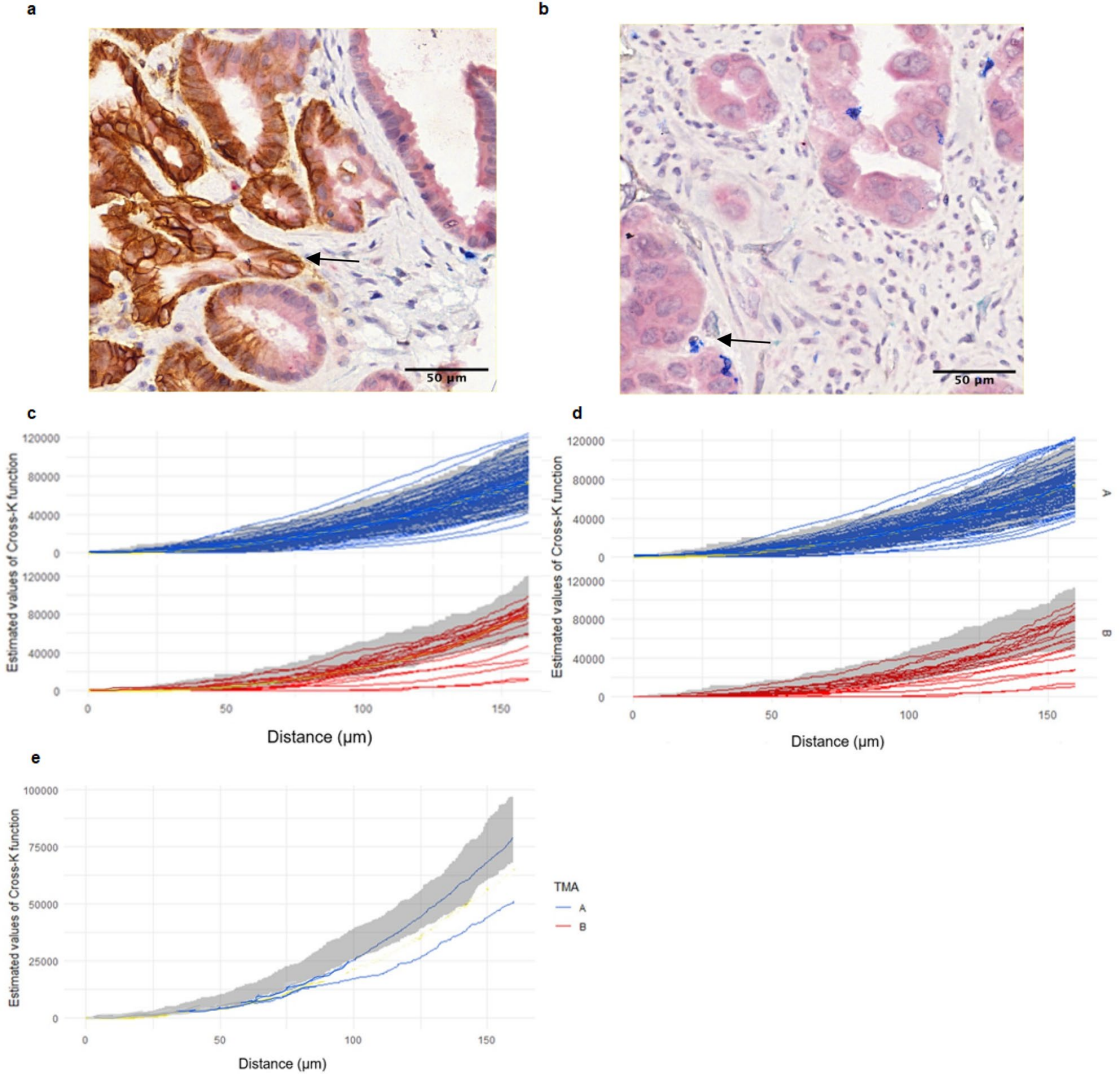
Staining images of CAIX and CD8 + and estimated cross-K functions of CAIX and CD8 + T cells with aggregate envelopes (gray area) from simulations by TMA and tissues. (**a**) Representative staining image of CAIX in brown. (**b**) Representative staining image of CD8 + in blue. (**c**) Cross-K functions plot for all COEUR. (**d**) Cross-K functions plot for stroma tissue, negative spatial association observed in TMA B, as shown in the plot 7 estimated cross-K functions (red lines) below the envelope. (**e**) Cross-K functions plot for tumor tissue, estimated cross-K functions not displayed for the density of CAIX and CD8 + below the threshold.

Given the discordant transcriptomic results between the four CCOC studies above, we hypothesized that a spatial relationship could provide a more meaningful explanation for the associations between hypoxic tumors and immune cell infiltration. To address this, immunostaining was performed using tissue microarrays obtained from the Canadian Ovarian Experimental Unified Resource (COEUR). The COEUR cohort consisted of two TMAs, TMA A and TMA B, each case represented by two cores for 183 and 218 cores respectively. Two representative images of tissues from TMA B show CAIX and CD8 + staining (Fig. 2A,B). A spatial relationship analysis was conducted by estimating the cross-K functions for CAIX and CD8 T cells for all of the cores along with the aggregated simulation envelopes, and these are presented separated by TMA A and B (Fig. 2C). A significant amount of heterogeneity in the spatial correlation patterns was observed in TMA A, and thus any trend in the spatial relationship between CD8 T cells and CAIX is masked by the heterogeneity across subjects. In TMA B there is an observable and clear signal, where there is a shift in the distribution of the cross-K functions estimated from the data and the Monte Carlo envelopes corresponding to independence (Fig. 2C; shaded grey regions). In particular, the distribution of estimated functions is shifted downwards relative to the Monte Carlo envelopes, indicating a trend towards a negative repulsion-like spatial association between CD8 T cells and CAIX covering a range from about 25–160 $\mathrm{\mu m}.$

Stratifying the spatial analysis by both TMA and tissue type, spatial patterns for stroma tissue that resemble the overall spatial patterns (Fig. 2D). Namely, the degree of subject variability in TMA A is too large to detect a pattern of spatial dependence between CD8 T cells and CAIX in stroma tissue; whereas, the analysis from TMA B indicates CAIX expression in the stroma is more likely to exhibit an immune cold phenotype with range from about 25–160 $\mathrm{\mu m}.$ Only two cores have CD8 + T cell density within tumor tissue that were sufficiently high to obtain stable Monte Carlo envelopes (Fig. 2E).

### Patient dependent differences in hypoxia and CD8 + T cell infiltration

Next, we asked whether the tumor and stroma associations between CAIX and CD8 + could be observed within a given patient core based on core-level summaries. Spearman correlation coefficients and associated bootstrap confidence interval analysis was performed on the COEUR dataset (Table 2). On TMA A there was a modest trend between CAIX and CD8 either overall (− 0.0677; − 0.2233, 0.0877) and after stratifying by tumor (0.02; − 0.1180, 0.1619) or stroma (− 0.0162; − 0.1731, 0.1382). In contrast, TMA B showed clear evidence of a negative relationship overall (− 0.3187; − 0.4480, − 0.1927) and after stratification to stroma (− 0.2956; − 0.4260, − 0.1679) but not to the tumor (− 0.08163; − 0.2125, 0.0479). Thus, the overall negative association between CD8 and CAIX on TMA B appears to arise from a negative association in the stroma.

**Table 2:** Spearman rank correlation and 95% Bootstrap confidence intervals for correlation between CAIX and CD8 + T cell counts for different tissue samples and stratified by tumor/stroma.

Spearman correlation and 95% Bootstrap confidence intervals				
TMA	A (n = 183)		B (n = 218)	
	− 0.0677 (− 0.2233, 0.0877)		− 0.3187 (− 0.4480, − 0.1927)	
Tissue	Tumor (n = 180)	Stroma (n = 183)	Tumor (n = 216)	Stroma (n = 218)
	0.0212 (− 0.1180, 0.1619)	− 0.0162 (− 0.1731, 0.1382)	− 0.08163 (− 0.2125, 0.0479)	− 0.2956 (− 0.4260, − 0.1679)

Model comparisons based on the AIC indicated that the negative binomial regression models were preferred uniformly over the zero-inflated Poisson regression models and thus we reported the results of the negative binomial regression models in Supplemental Table S1. We found evidence of a negative association between CD8 and CAIX overall (p-value = 0.0171) and in the stroma (p-value = 0.0050); whereas, no strong evidence of such an association could be detected in the tumor (p-value = 0.173). These negative associations between CD8 and CAIX were consistent across TMA A and B as there is no evidence of an interaction with TMA slide overall (p-value = 0.1325), in tumor (p-value = 0.165) and in stroma (p-value = 0.2442). The consistency of the negative association between CD8 and CAIX across both TMAs is summarized in Table 3 which presents 95% confidence intervals for the effect size of CAIX on CD8.

**Table 3:** 95% confidence intervals of the effect of CAIX on CD8 conditional on TMA from the negative binomial regression model.

Samples	TMA A	TMA B
All COEUR	(− 0.00164, − 0.00016)	(− 0.00286, − 0.00085)
Tumor	(− 0.00196, 0.01092)	(− 0.04937, 0.01316)
Stroma	(− 0.00199, − 0.00035)	(− 0.00302, − 0.00091)

### Survival analysis

The clinical characteristics and the computed intensities for CD8 and CAIX from the tumor tissue in the COEUR cohort is summarized in Supplemental Table S2. Kaplan–Meier survival curves and p-values from the log-rank test indicated that survival time was significantly different for participants with different levels of debulking, FIGO-stage, and family cancer history (Supplemental Fig. S3). Cases were categorized with respect to CD8 + T cells and CAIX expression based on 34th and 66th percentiles into low, moderate, and high groups for Kaplan–Meier survival curves. Accordingly, except for age, the lack of familial history, optimal debulking, early staging and tumor grade NA (not available) were found to be prognostic for improved overall survival (Supplemental Fig. S3).

Next, we used multivariable Cox proportional hazard models to examine the association between core-specific CAIX expression and CD8 + T cell infiltration and survival time, while adjusting for patient characteristics. For patients with multiple cores the average density of the biomarkers across cores was incorporated into the proportional hazard model. In conducting this analysis, examination of diagnostics suggested one participant whose data was extreme in the sense that its exclusion had an unusually sizeable impact on the results (Supplemental Tables S3, S4). The data from this participant was subsequently removed from the analysis and a leave-one-out analysis was also conducted to assess the stability of the results to the removal of the remaining subjects (Supplemental Fig. S4). The sensitivity analysis indicated stability for the remaining data. The results are presented in Supplemental Table S5.

After adjusting for the other variables, the CD8 density is positively related to survival (Supplemental Table S5, p-value 0.047) with an estimated hazard ratio of 0.974 for each unit increase in cell density (95% CI 0.950, 1.000). For comparison, the results from univariable models are presented in Supplemental Table S5 as well. A posthoc power analysis showed that the sample size of 154 subjects yields a power of 0.83 to detect the observed effects of CD8 density (which on the scale of log hazard ratio is − 0.026 for unit increase in CD8 density). The estimated relationship between the hazard ratio and CD8 density is further depicted in Supplemental Fig. S6. Model diagnostics are based on a Kolmogorov-type supremum test^30^ and presented in Supplemental Fig. S5 and Supplemental Tables S6, S7.

## Discussion

Hypoxia-mediated disruption in tumor metabolism is a hallmark of cancer. The changes imparted by hypoxia occur on multiple levels both within the tumor epithelium and surrounding host tissue including stroma and other cancer associated cells. Another crucial impact of an oxygen deprived tumor microenvironment is on antitumor immunity. Indeed, changes in the tumor vasculature and transcriptional metabolic responses to HIF-1 dependent gene expression can serve as a mechanical, nutritional, and functional barrier to immune cell infiltration. A number of pre-clinical studies have shown that tumor hypoxia is an upstream determinant of immune phenotypes^42,43^. That is, low oxygen is associated with immune cold tumors and may potentially explain why such tumors are resistant to immunotherapy. The precise mechanisms that trigger hypoxia-induced immune cell exclusion are not known. Moreover, there is no gold-standard method to assess hypoxic signatures especially in the scenario of managing patients who are undergoing standard of care for their cancer or correlative studies in patients who are participating in clinical trials. Given the varied ex vivo and in vivo techniques available to detect hypoxia and immune infiltration, we undertook this study to map the relationship between hypoxia and T cell exclusion in CCOC, a histotype that has been purported to exhibit features of hypoxia.

Several different transcriptional signatures of hypoxia have been applied to investigate the extent of hypoxia in surgically resected tissues. In general, a high hypoxic gene score is associated with an immune suppressive environment^37,38^. Almost universally, this hypoxia-immune relationship was prognostic for poor overall survival. These data imply that hypoxia is intrinsically linked to the extent of immune cell infiltration and this phenotype may explain in part the immune excluded feature of solid tumors. In contrast, our analysis did not reveal any evidence for an association between a hypoxia score and CD8. However, when CAIX, a well-established hypoxia marker was used to stratify the presence or absence of CD8, a strong positive association was observed in two of the four publicly available CCOC datasets. Although it is not clear why differences in the correlation between CAIX and were found between these cohorts, several reasons may explain these discordant associations. One of the biggest limitations is the relatively small number of CCOC cases the GSE6008 (n = 8) and GSE29450 (n = 10). Another consideration is that the GSE29450 study was conducted on laser capture micro-dissected tumor tissue. It should also be pointed out that the microarray platforms differed. Biologically acute decreases in oxygen functionally impair CD8 + T cells whereas chronic hypoxia promotes the decrease in cell survival. Indeed, neither [^18^F]EF5 or bulk transcriptomic analyses are able to capture the temporal cycles of hypoxia.

The central role of oxygen deprivation on CD8 + T cell responses remains a matter of debate. This is in part due to the lack of primary human studies examining the association between CAIX (and other hypoxia markers) and infiltration of T cells. However, the general consensus based on preclinical murine models and our own in vitro studies suggest that low oxygen is incompatible with CD8 + T cell survival and anti-tumor activity^11,44–46^ Mechanistically, hypoxia can promote a terminally exhausted phenotype through mitochondrial stress and dysfunction. As a result, T cells may be excluded from hypoxic micro-regions of the tumor. On the other hand, studies have shown that hypoxia-inducible factor activity and priming T cells under hypoxia can imparts enhanced anti-tumor function^14,47^. To some extent, the use of different hypoxia markers may explain these discrepancies. For instance, CAIX is a considered a stable marker of a prior hypoxia event, whereas HIF-1 is a labile and acute surrogate of hypoxia. Based on publicly available data, the hypoxia signature genes we employed showed a negative association with the extent of CD8 + T cell expression in two out of the four cohorts (Figs. S1 and S3). However, the two cohorts where the hypoxia signatures were found to be negatively associated with CD8 + T cells, a similar analysis of CAIX expression showed a discordant result. While the precise reason for this difference is not known, the aforementioned biological and technological limitations of assessing CAIX and hypoxia signature genes are likely part of this explanation. Future studies on larger cohorts may help clarify this issue.

Recent advances in multiplex immunostaining have facilitated direct spatial orientation of CD8 + T cell infiltration with hypoxia markers. We hypothesized that coupling the analysis between CAIX and CD8 + T cells in a spatial analysis may reveal a better topographic understanding of the spatially and regionally distinct organization of hypoxia in regions of the TME. Using the retrospective COEUR cohort, we employed spatially-resolved analysis of CD8 and CAIX expressing cells as marked point processes using geospatial analysis frameworks. As expected, there was marked heterogeneity within the tumor and stroma in the spatial relationships between these compartments of the TME. Some of the cores in the COEUR TMA demonstrated evidence of CD8 and CAIX co-clustering in the tumor. This is consistent with studies showing that CD8 + T cells can become functionally inhibited upon exposure to acute oxygen deprivation oxygen^8^. In other cores, CD8 and CAIX showed repulsion which may suggest that in some cases chronic exposure to hypoxia can impair the survival of CD8 + T cells. Future studies will need to consider the short- versus long-term effects of low oxygen on CD8 + T cells. The results showing that hypoxia may promote CD8 + T cell exclusion presented here in CCOC may be a more broadly occurring phenomenon as this feature is also observed in high-grade serous ovarian carcinoma^8^.

Although no clear relationship between these markers could be observed in the tumor, at the resolution of the stroma, there was an overall and spatially specific negative association between CD8 and CAIX. While this result is consistent with the idea that hypoxia leads to an immune cold phenotype, it is possible that in CCOC, the intensity and duration of hypoxia found in the stroma could represent the main compartmental impediment to CD8 + T cell survival. It is possible CD8 + T cells that are able to persist and survive in the hypoxic stroma may have a greater probability of infiltrating into the tumor epithelium. Interestingly, our multivariable analysis revealed that the presence of CD8 + T cells in the tumor was associated with improved outcome, when adjusting for CAIX expression and other patient characteristics. Thus, the outcome lends support to this model. However, formal validation whereby the migration of CD8 + T cells into subregions of the TME under different degrees of oxygen tension is necessary to validate this postulation.

Our multi-modality study underscores that hypoxia within the CCOC TME is common, and appears to exclude CD8 + T cells, thereby creating an immune cold phenotype. In situations where CD8 + T cells can survive the low oxygen levels in the stroma, infiltration into the tumor can in turn lead to improved outcome. Therefore, therapeutic strategies to improve CD8 + T cell may need to consider targeting the hypoxic stroma environment.

## Supplementary Information

Supplementary Information.

## Data Availability

The data that support the findings of this study are available from biobanquerrcancer.chum@ssss.gouv.qc.ca (COEUR Study) and the Cheryl Brown Outcomes Unit info@ovcare.ca (EF5 Study) but restrictions apply to the availability of these data, which were used under license for the current study, and so are not publicly available. Data are however available from the authors upon reasonable request and with permission from the above-mentioned sources.

## Abbreviations

CAIX: Carbonic anhydrase IX
CAR-T: Chimeric antigen receptor T cells
CCOC: Clear cell ovarian carcinoma
COEUR: Canadian ovarian experimental unified outcomes resource
CTL: Cytotoxic T lymphocyte
HIF-1α: Hypoxia inducible factor 1 alpha
NS: Normal saline
PET/CT: Positron emission tomography/ computed tomography
PH: Proportional hazards
ssGSEA: Single-sample gene set enrichment analysis
SUV: Standardized uptake values
T/M: Tumor-to-muscle activity ratio
TMAs: Tissue microarrays
TME: Tumor microenvironment
