# Supplementary material for: CD8 + T cell infiltration is associated with improved survival and negatively correlates with hypoxia in clear cell ovarian cancer
Source: Sci Rep. 2023 Apr 21;13:6530. doi: 10.1038/s41598-023-30655-3 (PMC10121667; doi:10.1038/s41598-023-30655-3)
Supplement: Supplementary file 1 — Supplementary Information. [file 41598_2023_30655_MOESM1_ESM.docx]

**Supplementary Material**

**CD8+ T cell infiltration is associated with improved survival and negatively correlates with hypoxia in clear cell ovarian cancer**

Nancy Guo^1^, Aijun Yang^1^, Fabiha Binte Farooq^2^, Shreena Kalaria^3^, Elena Moss^3^, Lindsay DeVorkin^3^, Mary Lesperance^1^, François Bénard^4^, Don Wilson^5^, Anna V. Tinker^6*^, Farouk S. Nathoo^1,*^, Phineas T. Hamilton^3,*^, Julian J. Lum^3,7,*^

^1^Department of Mathematics and Statistics, University of Victoria

^2^Department of Economics, University of Victoria

^3^Trev and Joyce Deeley Research Centre, BC Cancer

^4^Department of Radiology, University of British Columbia

^5^Functional Imaging, BC Cancer Vancouver

^6^Medical Oncology, BC Cancer Vancouver

^7^Department of Biochemistry and Microbiology, University of Victoria

^*^ These authors contributed equally to the manuscript. Correspondence concerning this article should be addressed to Julian J. Lum, Deeley Research Centre, BC Cancer – Victoria, 2410 Lee Avenue, 3^rd^ Floor, Victoria, BC, V8R 6V5. E-mail: [jjlum@bccancer.bc.ca](mailto:jjlum@bccancer.bc.ca). Phineas T. Hamilton, Deeley Research Centre. Email: phin.hamilton@gmail.com. Farouk S. Nathoo, Department of Mathematics and Statistics, University of Victoria, PO BOX 1700 STN CSC, Victoria, BC, V8W 2Y2. Email: nathoo@uvic.ca. Anna V. Tinker, BC Cancer. 600 West 10th Ave Vancouver BC Email: atinker@bccancer.bc.ca


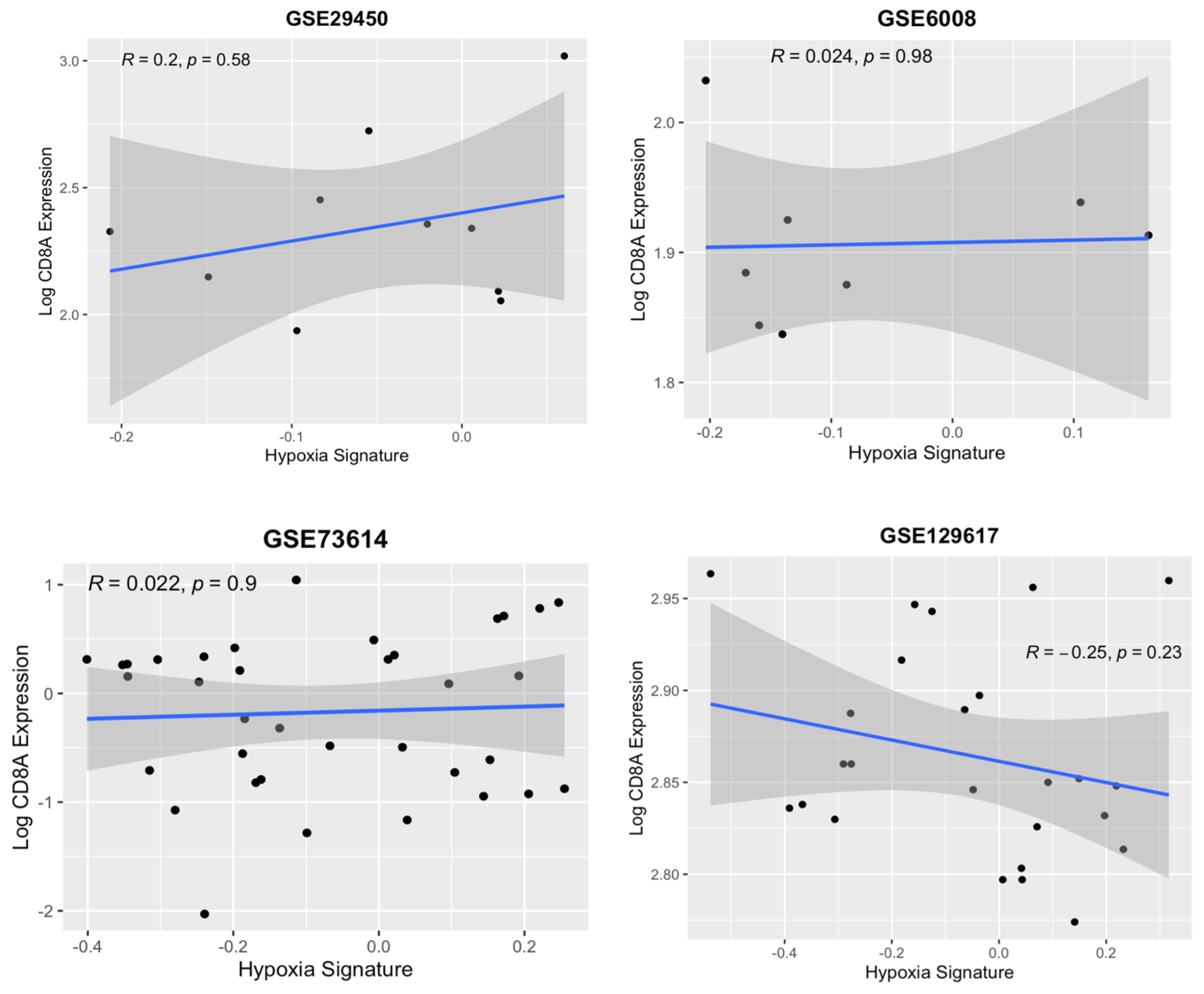
Figure S1 Scatter plot and fitted regression lines demonstrating the relationship between Log CD8 expression and hypoxia gene signature in four NCBI studies.


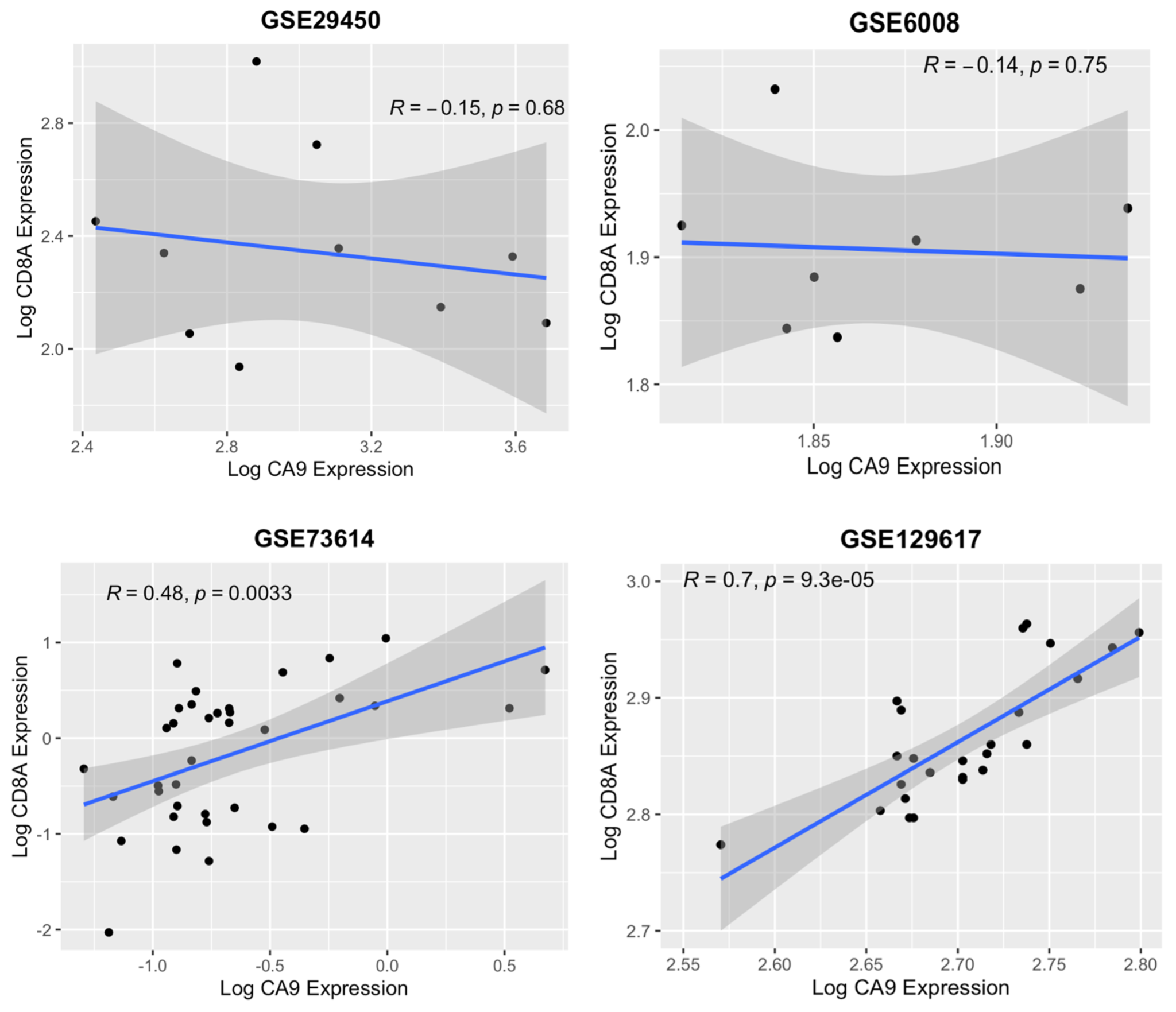
Figure S2 Scatter plots and fitted regression lines demonstrating the relationship between Log CD8A expression and Log CAIX (CA9) expression in four NCBI studies.


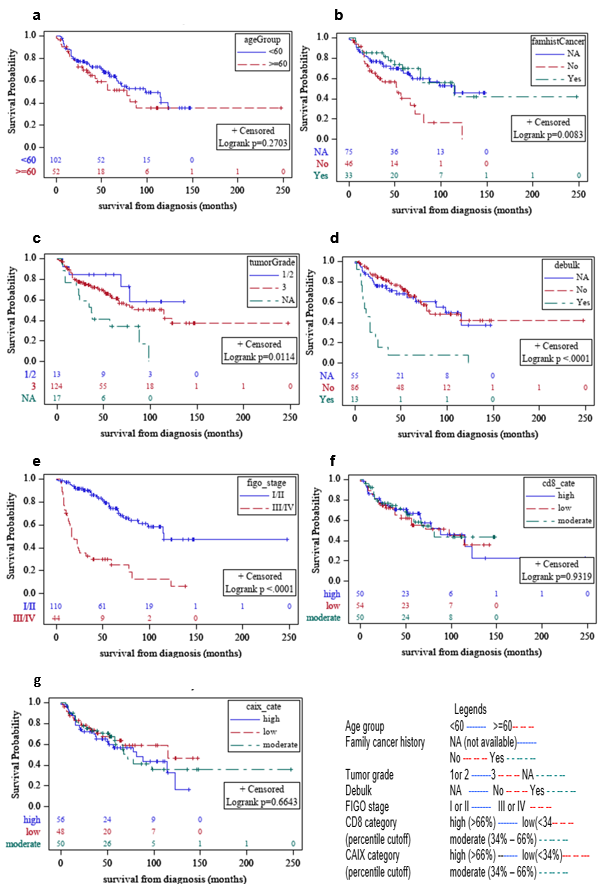


Figure S3 Kaplan-Meier survival curves stratified by study categorical variables


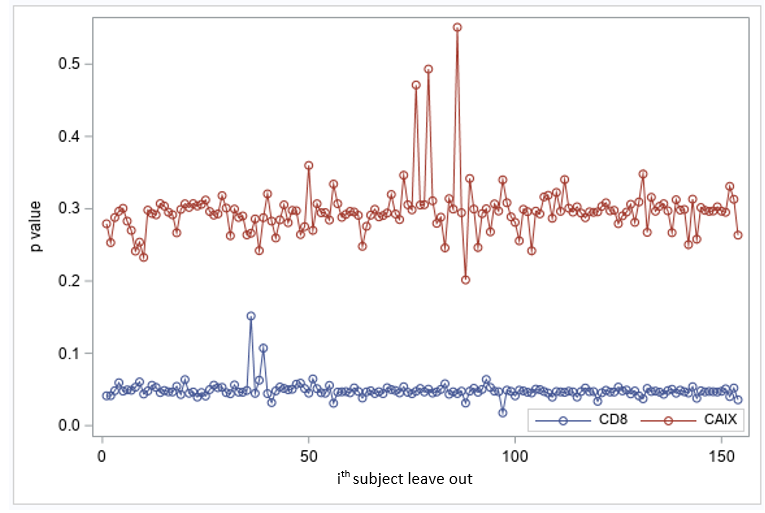


Figure S4 p-values obtained in the Cox proportional hazards model as each of the 154 subjects in the sample is removed. The red curve depicts the p-values for CAIX while the blue curve depicts the corresponding p-values for CD8. The average leave-one-out p-value for CD8 is 0.049 while the average for CAIX is 0.294.


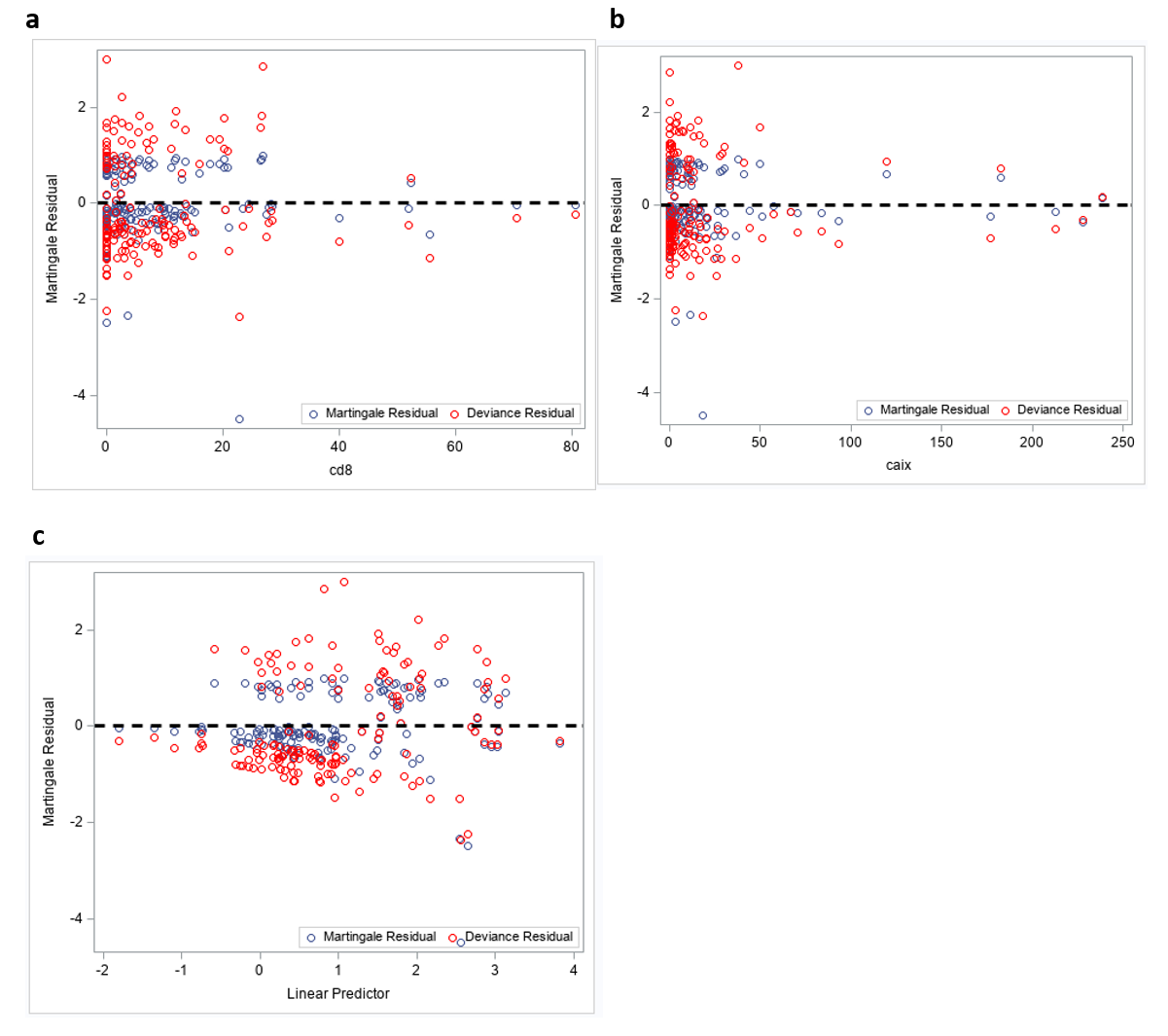


Figure S5 Martingale and deviance residuals plots for checking linearity of covariates. **a** Martingale and deviance residuals vs CD8 **b** Martingale and deviance residuals vs CAIX **c** Martingale and deviance residuals vs linear predictor scores


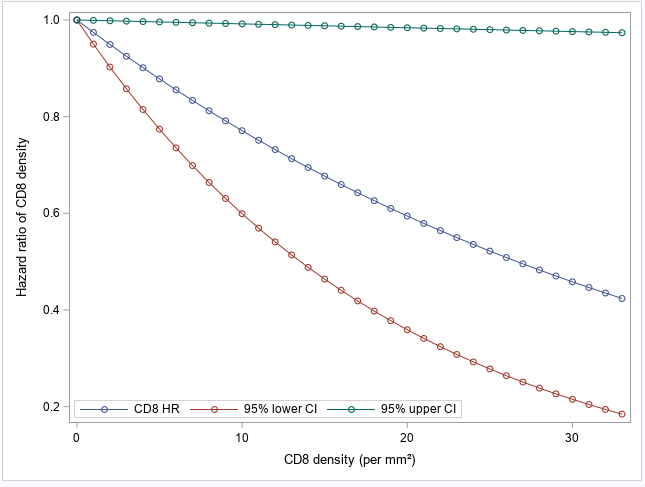


Figure S6 Predicted hazard ratio of CD8 density with 95% confidence intervals with other categorical covariates taking the reference level (or zero for continuous covariate).


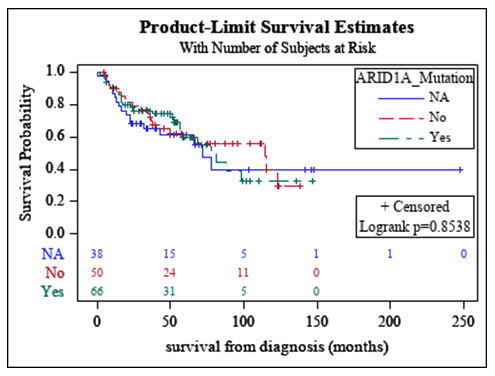


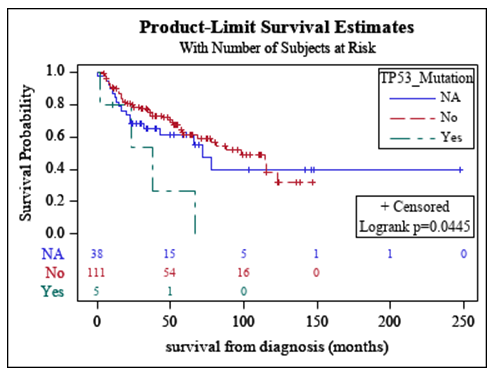


Figure S7 Kaplan-Meier survival curves stratified by ARID1A and TP53 mutations with Log rank test p-values.


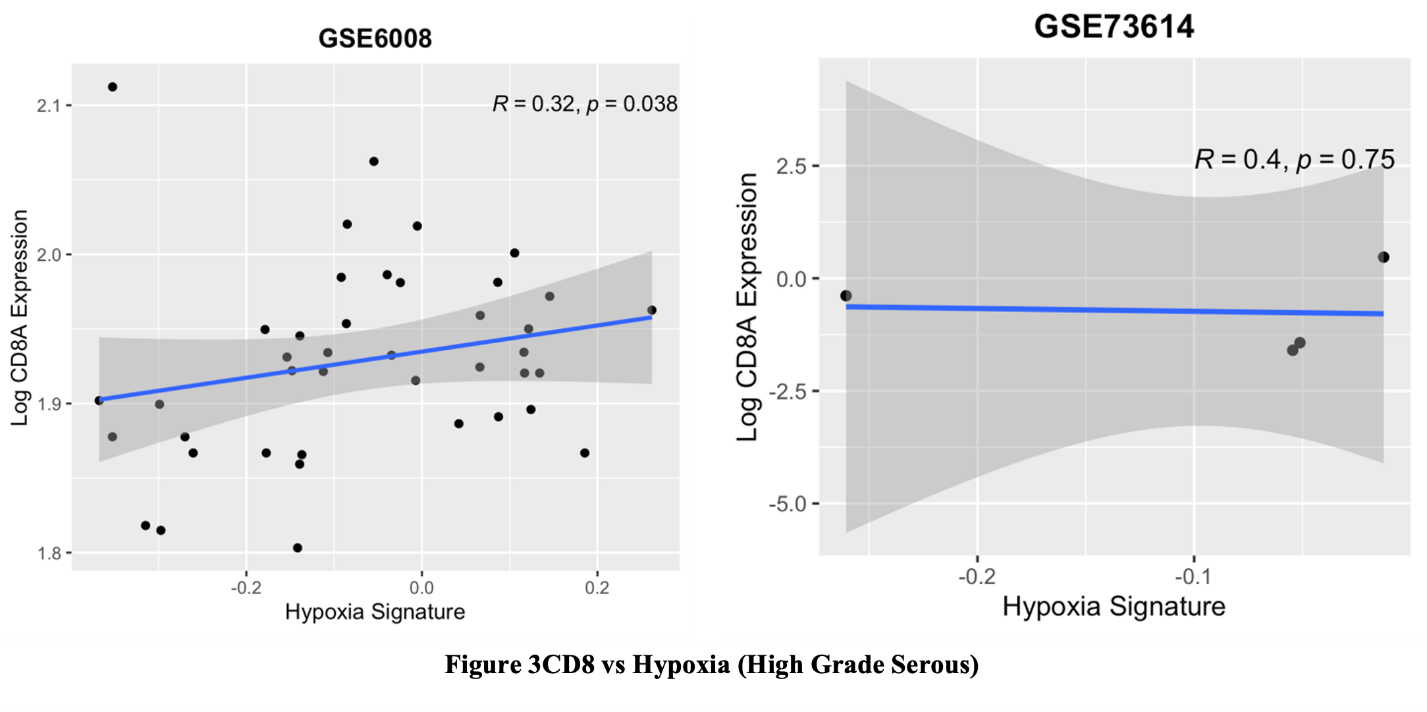


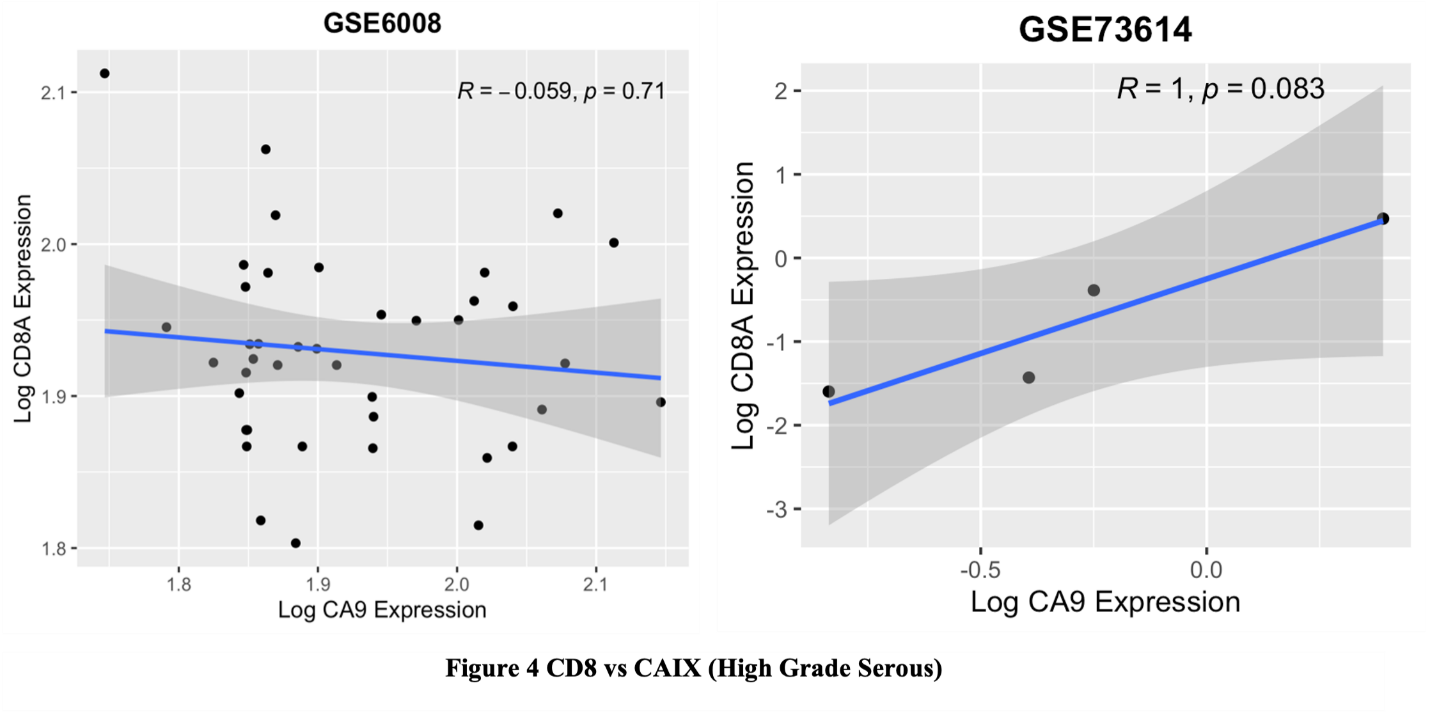


Figure S8 Scatter plots and fitted regression lines demonstrating the relationship between (i) top row - Log CD8A expression and hypoxia gene signature (ii) bottom row – Log CD8A and Log CA9 expression for High Grad Serous samples in two NCBI studies.

Table S1 Estimated regression coefficients obtained from the negative binomial model for different tissue samples

| **Covariates** | **Estimate** | **95% confidence intervals** | **p-value** |
| --- | --- | --- | --- |
| **All COEUR** |  |  |  |
| Intercept | 4.2934 | (4.0921, 4.4946) | 0.0000 |
| CAIX | -0.00090 | (-0.00164, -0.00016) | 0.0171 |
| TMA B | -0.7221 | (-0.9901, -0.4541) | 0.0000 |
| CAIX*TMA B | -0.00096 | (-0.0022, 0.0003) | 0.1325 |
|  |  |  |  |
| **Tumor** | | | |
| Intercept | 0.9024 | (0.6470, 1.1578) | 0.0000 |
| CAIX | 0.0045 | (-0.0020, 0.0109) | 0.173 |
| TMA B | 0.1354 | (-0.2097, 0.4806) | 0.442 |
| CAIX*TMA B | -0.0226 | (-0.0545, 0.0093) | 0.165 |
|  |  |  |  |
| **Stroma** | | | |
| Intercept | 4.2669 | (4.0629, 4.4710) | 0.0000 |
| CAIX | -0.0012 | (-0.00199, -0.00035) | 0.0050 |
| TMA B | -0.7806 | (-1.0526, -0.5086) | 0.0000 |
| CAIX*TMA B | -0.0008 | (-0.0021, 0.0005) | 0.2442 |

Table S2 Baseline characteristics and CD8 and CAIX density

| Variables | Total (N=154) | Alive (N=89) | Dead (N=65) |
| --- | --- | --- | --- |
| Age at Diagnosis |  |  |  |
| Mean (95%CI) | 56.4±9.5 | 56.3±8.7 | 56.7±10.6 |
| Min–Max | 35 – 80 | 35 – 78 | 35 – 80 |
| Median (IQR) | 55 (50 – 63) | 55 (51 – 62) | 55 (49 – 65) |
| Family Cancer History |  |  |  |
| NA | 75 (48.7) | 47 (52.8) | 28 (43.1) |
| No | 46 (29.9) | 21 (23.6) | 25 (38.5) |
| Yes | 33 (21.4) | 21 (23.6) | 12 (18.5) |
| Tumor Grade |  |  |  |
| 1/2 | 13 (8.4) | 9 (10.1) | 4 (6.2) |
| 3 | 124 (80.5) | 76 (85.4) | 48 (73.8) |
| NA | 17 (11.0) | 4 (4.5) | 13 (20.0) |
| Debulking |  |  |  |
| NA | 55 (35.7) | 34 (38.2) | 22 (32.3) |
| No | 86 (55.8) | 55 (61.8) | 31 (47.7) |
| Yes | 13 (8.4) |  | 13 (20.0) |
| Figo Stage |  |  |  |
| I/II | 110 (71.4) | 79 (88.8) | 31 (47.7) |
| III/IV | 44 (28.6) | 10 (11.2) | 34 (52.3) |
| CD8 density cells/mm² |  |  |  |
| Mean (95%CI) | 8.56 (0.00–33.77) | 9.26 (0.00–38.20) | 7.60 (0.00–26.61) |
| Min–Max | 0.00–80.68 | 0.00–80.68 | 0.00–52.40 |
| Median (IQR) | 3.76 (0.00–11.61) | 4.12 (0.00–10.77) | 3.71 (0.00–11.92) |
| CAIX density cells/mm² |  |  |  |
| Mean (95%CI) | 17.49 (0.00–95.24) | 14.90 (0.00–79.41) | 21.03 (0.00–114.02) |
| Min–Max | 0.0–238.48 | 0.00–213.01 | 0.00–238.48 |
| Median (IQR) | 3.76 (0.00–15.56) | 2.94 (0.00–13.91) | 6.65 (0.73–15.56) |

Table S3 Results of survival analysis (Cox PH) obtained from total sample of 155 subjects

| **Covariates** | **Estimate** | **Std Error** | **P value** | **Hazard Ratio (HR)** | **HR Lower CL** | **HR Upper CL** | **P-value** |
| --- | --- | --- | --- | --- | --- | --- | --- |
| Age group (>=60 vs <60) | 0.49659 | 0.27081 | 0.0667 | 1.643 | 0.966 | 2.794 | 0.0667 |
| Tumor grade (3 vs 1/2) | 0.4906 | 0.54784 | 0.3705 | 1.633 | 0.558 | 4.78 | 0.3705 |
| (NA vs 1/2) | 1.35248 | 0.58658 | 0.0211 | 3.867 | 1.225 | 12.209 | 0.0211 |
| figo_stage (III/IV vs I/II) | 1.58936 | 0.30449 | <.0001 | 4.901 | 2.698 | 8.901 | <0.0001 |
| debulk (Yes vs No) | 0.95445 | 0.40173 | 0.0175 | 2.597 | 1.182 | 5.708 | 0.0175 |
| (NA vs No) | 0.46687 | 0.37975 | 0.2189 | 1.595 | 0.758 | 3.357 | 0.2189 |
| Fam hist cancer (Yes vs No) | -0.31945 | 0.41319 | 0.4394 | 0.727 | 0.323 | 1.633 | 0.4394 |
| (NA vs No) | -0.55471 | 0.3944 | 0.1596 | 0.574 | 0.265 | 1.244 | 0.1596 |
| CD8 density (cells/mm^2^) | -0.02409 | 0.01234 | 0.0509 | 0.976 | 0.953 | 1 | 0.0509 |
| CAIX density (cells/mm^2^) | 0.00448 | 0.00122 | 0.0002 | 1.004 | 1.002 | 1.007 | **0.0002** |

Table S4 Results of survival analysis (Cox PH) obtained from 154 subjects with the one subject causing instability removed

| **Covariates** | **Estimate** | **Std Error** | **P value** | **Hazard Ratio (HR)** | **HR Lower CL** | **HR Upper CL** | **P-value** |
| --- | --- | --- | --- | --- | --- | --- | --- |
| Age group (>=60 vs <60) | 0.51609 | 0.27497 | 0.0605 | 1.675 | 0.977 | 2.872 | 0.0605 |
| Tumor grade (3 vs 1/2) | 0.48059 | 0.54746 | 0.38 | 1.617 | 0.553 | 4.728 | 0.3800 |
| (NA vs 1/2) | 1.31952 | 0.5887 | 0.025 | 3.742 | 1.18 | 11.862 | 0.0250 |
| figo_stage (III/IV vs I/II) | 1.59992 | 0.30741 | <.0001 | 4.953 | 2.711 | 9.047 | <0.0001 |
| debulk (Yes vs No) | 1.01561 | 0.42011 | 0.0156 | 2.761 | 1.212 | 6.29 | 0.0156 |
| (NA vs No) | 0.44759 | 0.3821 | 0.2414 | 1.565 | 0.74 | 3.309 | 0.2414 |
| Fam hist cancer (Yes vs No) | -0.27923 | 0.42121 | 0.5074 | 0.756 | 0.331 | 1.727 | 0.5074 |
| (NA vs No) | -0.49874 | 0.40862 | 0.2223 | 0.607 | 0.273 | 1.353 | 0.2223 |
| CD8 density (cells/mm^2^) | -0.02586 | 0.01302 | 0.047 | 0.974 | 0.95 | 1 | 0.0470 |
| CAIX density (cells/mm^2^) | 0.00308 | 0.00294 | 0.2939 | 1.003 | 0.997 | 1.009 | **0.2939** |

Table S5 Cox Proportional Hazard Regression Analysis on Survival Time (Tumor Tissue)

| **Covariates** | **Univariable** | | **Multivariable** | |
| --- | --- | --- | --- | --- |
|  | **Hazard Ratio(95% CI)** | ***p*** | **Hazard Ratio(95% CI)** | ***p*** |
| **Age (>=60 vs <60 y)** | 1.326 ( 0.800 , 2.199 ) | 0.274 | 1.675 ( 0.977 , 2.872 ) | 0.061 |
| **FIGO Stage** (III/IV vs I/II) | 5.291 ( 3.217 , 8.701 ) | <0.0001 | 4.953 ( 2.711 , 9.047 ) | <0.0001 |
| **Debulking** |  |  |  |  |
| Yes vs No | 6.023 ( 3.100 , 11.702 ) | <0.0001 | 2.761 ( 1.212 , 6.290 ) | 0.016 |
| NA vs No | 1.138 ( 0.653 , 1.982 ) | 0.648 | 1.565 ( 0.740 , 3.309 ) | 0.241 |
| **Tumor Grade** |  |  |  |  |
| 3 vs 1/2 | 1.635 ( 0.588 , 4.546 ) | 0.346 | 1.617 ( 0.553 , 4.728 ) | 0.380 |
| NA vs 1/2 | 3.687 (1.198 , 11.350 ) | 0.023 | 3.742 ( 1.180 , 11.862 ) | 0.025 |
| **Family Cancer History** |  |  |  |  |
| Yes vs No | 0.418 ( 0.208 , 0.841 ) | 0.015 | 0.756 ( 0.331 , 1.727 ) | 0.507 |
| NA vs No | 0.474 ( 0.273 , 0.824 ) | 0.008 | 0.607 ( 0.273 , 1.353 ) | 0.222 |
| **CD8 density cells/mm²** | 0.990 ( 0.970 , 1.010 ) | 0.324 | 0.974 ( 0.950 , 1.000 ) | 0.047 |
| **CAIX density cells/mm²** | 1.005 ( 0.999 , 1.010 ) | 0.090 | 1.003 ( 0.997 , 1.009 ) | 0.294 |

Table S6 Supremum test for functional form of CD8 and CAIX with 1000 replications

| **Covariates** | | **Maximum absolute Value** |  | ***P-*value** |
| --- | --- | --- | --- | --- |
| CD8 |  | 4.2068 |  | 0.706 |
| CAIX |  | 5.4849 |  | 0.505 |

Table S7 Supremum test for proportional hazards assumption with 1000 replications

| **Covariates (level)** | | **Maximum absolute value** |  | ***P-*value** |
| --- | --- | --- | --- | --- |
| Age group (>60) | | 0.604 |  | 0.706 |
| Tumor grade (3) | | 1.160 |  | 0.702 |
| Tumor grade (NA) | | 1.431 |  | 0.470 |
| figo stage (III/IV) | | 1.810 |  | 0.003 |
| debulk (Yes) | | 1.365 |  | 0.100 |
| debulk(NA) | | 0.759 |  | 0.821 |
| Fam hist cancer (Yes) | | 0.789 |  | 0.704 |
| Fam hist cancer (NA) | | 1.098 |  | 0.588 |
| CD8 |  | 0.747 |  | 0.411 |
| CAIX |  | 1.163 |  | 0.180 |

Table S8 – Hallmark genes from MSigDB used to compute the hypoxia score using GSVA

"ACKR3" , "ADM" , "ADORA2B", "AK4" , "AKAP12" , "ALDOA", "ALDOB" ,"ALDOC" , "AMPD3" , "ANGPTL4", "ANKZF1", "ANXA2" , "ATF3" , "ATP7A", "BCAN" , "BCL2", "BGN" , "BHLHE40" , "BNIP3L", "BRS3" , "BTG1" , "CA12", "CASP6" , "CAV1" , "CCNG2" , "CDKN1A" , "CDKN1B", "CDKN1C", "CDKN1C", "CHST2", "CHST3" , "CITED2" , "COL5A1", "CP" , "CSRP2", "CXCR4" , "DCN", "DDIT3", "DDIT4", "DPYSL4", "DTNA" , "DUSP1", "EDN2", "EFNA1", "EFNA3", "EGFR", "ENO1", "ENO2", "ENO3", "ETS1", "EXT1" , "F3" , "FAM162A" , "FBP1", "FOS", "FOSL2", "FOXO3" , "GAA", "GALK1", "GAPDH", "GAPDHS", "GBE1" , "GCK", "GCNT2", "GCNT2", "GLRX", "GPC1" , "GPC3", "GPC4" , "GPI" , "GPI", "GRHPR", "GYS1", "HAS1" , "HDLBP" , "HEXA", "HK1" , "HK2", "HMOX1", "HOXB9" , "HS3ST1" , "HSPA5", "IDS" , "IER3", "IER3" , "IER3" , "IER3", "IER3", "IER3" , "IGFBP1" "IGFBP3",, "IL6" "ILVBL", "INHA" , "IRS2" , "ISG20", "JMJD6", "JUN" , "KDELR3" "KDM3A" "KIF5A" "KLF6" "KLF7" "KLHL24", "LALBA", "LDHA" , "LDHA", "LDHC" , "LOX", "LXN" , "MAFF", "MAP3K1" , "MIF" , "MIF", "MT1E", "MT2A" , "MXI1", "MYH9" , "NAGK", "NCAN" , "NDRG1", "NDST1" , "NDST2" "NEDD4L" "NFIL3" "NR3C1" "P4HA1" "PAM", "PCK1" , "PDGFB", "PDK1", "PDK3", "PFKFB3" , "PFKL" , "PFKP", "PGAM2", "PGF", "PGK1", "PGM1" , "PHKG1", "PIM1", "PKLR" , "PKLR", "PKP1" , "PLAC8", "PLAUR", "PLIN2", "PNRC1", "PPARGC1A", "PPFIA4" , "PPP1R15A", "PPP1R3C", "PRKCA", "PYGM" , "RBPJ" , "RORA" , "RRAGD", "S100A4", "SAP30" ,"SCARB1", "SDC2" , "SDC3" , "SDC4", "SELENBP1", "SERPINE1", "SIAH2" , "SLC25A1" , "SLC2A1", "SLC2A3", "SLC2A5", "SLC37A4", "SLC37A4", "SLC6A6", "SRPX", "STC1", "STC2", "SULT2B1", "TES" , "TGFB3", "TGFBI", "TGM2", "TIPARP", "TKTL1" , "TMEM45A", "TNFAIP3", "TPBG" , "TPBG" , "TPD52", "TPI1", "TPST2", "UGP2", "VEGFA", "VHL", "VLDLR", "WSB1", "XPNPEP1", "ZFP36", "ZNF292"
